# Impact of Heavy Metal Exposure on *Mytilus galloprovincialis* Spermatozoa: A Metabolomic Investigation

**DOI:** 10.3390/metabo13080943

**Published:** 2023-08-13

**Authors:** Gennaro Lettieri, Carmela Marinaro, Rosaria Notariale, Pasquale Perrone, Martina Lombardi, Alessio Trotta, Jacopo Troisi, Marina Piscopo

**Affiliations:** 1 Department of Biology, University of Naples Federico II, Via Cinthia, 21, 80126 Naples, Italy; 2 Department of Biology and Evolution of Marine Organisms, Stazione Zoologica Anton Dohrn, Villa Comunale 1, 80121 Naples, Italy; 3 Department of Precision Medicine, School of Medicine, University of Campania “Luigi Vanvitelli”, Via Luigi de Crecchio, 80138 Naples, Italy; 4 Theoreo S.R.L.—Spin-off Company of the University of Salerno, 84098 Montecorvino Pugliano (SA), Italy

**Keywords:** spermatozoa, metabolomics, heavy metals, *Mytilus galloprovincialis*

## Abstract

Metabolomics is a method that provides an overview of the physiological and cellular state of a specific organism or tissue. This method is particularly useful for studying the influence the environment can have on organisms, especially those used as bio-indicators, e.g., *Mytilus galloprovincialis*. Nevertheless, a scarcity of data on the complete metabolic baseline of mussel tissues still exists, but more importantly, the effect of mussel exposure to certain heavy metals on spermatozoa is unknown, also considering that, in recent years, the reproductive system has proved to be very sensitive to the effects of environmental pollutants. In order to fill this knowledge gap, the similarities and differences in the metabolic profile of spermatozoa of mussels exposed to metallic chlorides of copper, nickel, and cadmium, and to the mixture to these metals, were studied using a metabolomics approach based on GC–MS analysis, and their physiological role was discussed. A total of 237 endogenous metabolites were identified in the spermatozoa of these mussel. The data underwent preprocessing steps and were analyzed using statistical methods such as PLS-DA. The results showed effective class separation and identified key metabolites through the VIP scores. Heatmaps and cluster analysis further evaluated the metabolites. The metabolite-set enrichment analysis revealed complex interactions within metabolic pathways and metabolites, especially involving glucose and central carbon metabolism and oxidative stress metabolism. Overall, the results of this study are useful to better understand how some pollutants can affect the specific physiological functions of the spermatozoa of this mussel, as well as for further GC–MS-based metabolomic health and safety studies of marine bivalves.

## 1. Introduction

The use of biomarkers for the assessment of the effects of exposure to chemical contaminants in the environment has been increasingly widespread in recent years [1,2,3]. Heavy metals are important contaminants in the pollution of the marine environment [4]. Benthonic organisms, and in particular bivalves, are extensively utilized as sentinel species in coastal environment monitoring [3,5,6]. Because mussels are sedentary organisms that feed on filter-feeding substances, they are exposed to large amounts of chemical pollutants. As a result, these organisms tend to bio-accumulate and magnify pollutants. They are therefore extremely useful in reflecting changes in pollution levels [7]. Heavy metals can bind to vital biomolecules, such as proteins and enzymes, altering enzyme activities, and leading to cellular dysfunction. Furthermore, heavy metals can produce reactive oxygen species (ROS), which can lead to the oxidative damage of cellular components, like lipids, proteins, and DNA. A relevant aspect to be considered is the reprotoxic effects of heavy metals on marine invertebrates. In the literature, this aspect has been partially explored and, in most studies, the impact of individual metals, as in the case of *Mytilus galloprovincialis* (*M. galloprovincialis*), has commonly been considered [8,9]. However, different heavy metals may act synergistically or antagonistically, increasing or decreasing their effects with respect to individual metals (manuscript under review into Chemico–Biological Interactions). An increasing number of techniques that measure the biological effects of pollutants have been developed in these programmes. However, traditional biomarkers appear to be more affected by the physiological state of mussels than by pollution [10,11], which has raised the need to search for new biological indicators of exposure to chemical pollutants. In this broader overview, ‘omics’ approaches such as genomics, transcriptomics, proteomics or metabolomics in environmental toxicology are particularly useful. (Manganese toxicity in soil for *Eisenia fetida* and *Enchytraeus crypticus*). ‘Omics’ technology has the potential to give new and more in-depth insights into the mode of action of pollutants, and this can contribute to a more comprehensive understanding of their environmental risk. Metabolomics is the study of the profile of low molecular weight endogenous metabolites of an organism that can be simultaneously detected and measured in small samples of biological tissue or fluid [12], with the objective of providing an overview of the metabolic condition of the biological system being examined. Environmental metabolomics is an approach to examine the metabolic responses of an organism to natural and anthropogenic stressors in its environment [13]. This approach examines changes in the metabolite concentrations, the precursors and products of enzyme activity, and aims to link these variations to biological function and/or regulation [12]. This approach has the potential to identify metabolites related to biochemical processes, providing insights into pathways disrupted by environmental stressors like pollutants, starvation, and pathogens [13,14,15,16,17]. Glycogen represents the primary energy storage molecule in mussels and is generally employed as a measure of the mussels’ condition in response to environmental stressors [18,19,20,21]. Previous studies conducted on marine bivalves reported the use of the metabolomics to characterise the metabolic responses and toxicity of specific contaminants [22,23,24]. For example, it has been shown that lead exposure can cause neurotoxicity, disturbances in energy metabolism, alterations in osmotic regulation, or lipid metabolism [25,26]. In the present work, we used GC–MS-based metabolomics, in order to evaluate the metabolomic changes in spermatozoa of *M. galloprovincialis* after 24 h exposure to three heavy metals individually (copper, nickel, and cadmium) in the form of chlorides, at doses of 15 µM, 15 µM, and 1.5 µM, respectively, and their mixture. These doses were used because they had previously been tested in our experiments and were the ones that showed the most relevant effects for other analyses also relating to the reproductive system of *M. galloprovincialis* [9,27,28,29,30]. GC–MS-based metabolomics, when associated with pattern recognition techniques, is capable of detecting subtle differences in the metabolome of similar samples and has the potential to elucidate the interactions between organisms and environment. In the present work, we evaluated the metabolomic changes in spermatozoa of this organism after 24 h of exposure to three heavy metals individually (copper, nickel, and cadmium) in the form of chlorides, at doses of 15 µM, 15 µM, and 1.5 µM, respectively, and their mixture. We have previously tested these doses for other aspects relating to the reproductive system of *M. galloprovincialis*, and they showed the greatest effects [9,27,28]. Differences and similarities in terms of qualitative and quantitative changes in their metabolic profiles, associated with the main physiological functions of these cells under investigation, were therefore analysed. The results of this study provide new insights into metabolite changes in spermatozoa and will be a basis for further investigations to better understand the toxicity of these metals on these cells.

## 2. Materials and Methods

### 2.1. Ethical Statement

This study was carried out on the marine invertebrate *M. galloprovincialis* (La-marck, 1819). This species is not protected by any environmental authority in Italy. This study was conducted in strict compliance with European (Directive 2010/63) and Italian (Legislative Decree 116/1992) legislation on the care and use of scientific animals.

### 2.2. Exposure of Mussels

Specimens provided by Eurofish Napoli S.R.L. Bacoli (Campania region), with an average shell size of 4.95 ± 0.17 cm and mixed sex, were selected to study the effects of heavy metals on *M. galloprovincialis*. The mussels were exposed to different doses of metals as reported in Piscopo et al. 2016 [31]: 15 µM, 15 µM, and 1.5 µM; nickel, copper, and cadmium, respectively, and their mixture at the same concentrations of single metals. Briefly, *M. galloprovincialis* specimens were exposed in plastic tanks measuring 36 cm × 22 cm× 22 cm. The tanks contained 6 L of 33‰ artificial seawater (ASW). Each L of ASW contained 29.2 g NaCl, 0.60 g KCl, 1.2 g MgCl_2_, 0.20 g NaHCO_3_, and 1.08 g CaCl_2_. 15 mussels were placed in each tank for 24 h at 18 ± 1 °C. The oxygen level and the temperature of the tanks were checked at regular intervals, and after 12 h, the water and the metal salts were changed. The experiments were carried out in February and March 2023. A tank containing only ASW, as described by Lettieri et al. 2019 [32], was used as a control (unexposed mussels).

### 2.3. Processing and Sampling of Spermatozoa

The mussels were opened with a knife after exposure to the various heavy metals tested. The soft tissues were left intact during this operation. The gonads were left in 500 µL of ASW for 5 min at 16 °C to facilitate the release of gametes. The sex of mussels was determined by optic microscope 40×. The gonads were then left in the same tube for 1 h. A further 500 µL of ASW was added to allow the release of all spermatozoa. The AWS containing spermatozoa was centrifuged at 2000× *g* for 1 min at 4 °C to remove debris. The supernatants obtained were then centrifuged at 9000× *g* for 10 min, and the collected spermatozoa (200 µL) were stored at −80 °C.

#### 2.3.1. Metabolite Extraction, Purification and Derivatization

The extraction, purification, and derivatization of the metabolome were conducted using the MetaboPrep GC kit (manufactured by Theoreo S.R.L, Montecorvino Pugliano [SA], Italy), according to the manufacturer’s instructions. In summary, a small volume of 50 µL serum was carefully transferred into a microcentrifuge tube, along with the extraction solution and internal standard. The tubes were vigorously mixed at a speed of 1250 rotations per minute (rpm) for half an hour. Afterwards, the solution underwent centrifugation at a temperature of 4 °C and a high speed of 16,000 rpm for 5 min. From the resulting mixture, 200 µL of the clear liquid on top (supernatant) was moved to a different microcentrifuge tube containing a purification solution. The new tube was then briefly mixed at 1250 rpm for half a minute and subjected to centrifugation once again at 16,000 rpm and 4 °C. Subsequently, 175 µL of the clear liquid (supernatant) was transferred to a glass vial and promptly frozen at −80 °C until it was freeze-dried overnight.

To prepare the freeze-dried samples for further analysis, the metabolites within them underwent derivatization using a two-step process. Initially, 50 μL of methoxylamine hydrochloride in pyridine was added to the freeze-dried samples and mixed at 1200 rpm for a duration of 90 min. Then, 25 µL of a derivatization solution containing N,O-Bis(trimethylsilyl)trifluoroacetamide (BSTFA) and trimethylchlorosilane (TMCS) was added to the mixture. The vials were once again mixed at 1200 rpm for an additional 90 min. The resulting derivatized metabolites (75 µL) were transferred to a vial insert with a capacity of 100 µL to facilitate injection into an autosampler. Prior to injection into the GC–MS instrument, the vials were centrifuged for 5 min at 16,000 rpm at 4 °C.

#### 2.3.2. GC-MS Analysis

Subsamples measuring 2 µL of the derivatized solution were introduced into the GC–MS system (consisting of a GC-2010 Plus gas chromatograph coupled to a 2010 Plus single quadrupole mass spectrometer; Shimazu Corp., Kyoto, Japan). Chromatographic separation was accomplished using a CP-Sil 8 CB fused silica capillary GC column, with dimensions of 30 m × 0.25 mm and a film thickness of 1.00 µM, obtained from Agilent (Agilent, J&W Scientific, Folsom, CA, USA). The carrier gas employed was helium. The temperature of the oven initially set at 100 °C was maintained for a duration of 1 min, following which it was raised at a rate of 6 °C per minute until reaching a final temperature of 320 °C. This final temperature was maintained for an additional 2.33 min. To ensure a constant linear velocity of 39 cm/s, the gas flow was adjusted accordingly, while the split flow was maintained at a ratio of 5:1. Operating in the electron impact mode at an energy of 70 eV, the mass spectrometer conducted a full scan analysis in the range of 35–600 *m*/*z*, with a scan velocity of 3333 amu/s and a solvent delay of 5 min. The entire GC program lasted for a total duration of 40 min.

#### 2.3.3. Metabolite Identification

Metabolite identification was conducted following the methodology of Troisi et al. [33,34,35]. Briefly, the identification of untargeted metabolites involved a comparison of the mass spectrum of each peak with the NIST-2014 library collection (NIST, Gaithersburg, MD, USA). For this comparison, a maximum tolerance of 10 for the linear retention index difference was set, while the minimum matching spectra library search was set at 85% (representing level 2 identification as per the Metabolomics Standards Initiative [MSI]) [36], whenever feasible. Metabolites that did not meet these criteria were classified as unknown, following MSI level 4 guidelines. The signals per sample generated by the combination of gas chromatography and mass spectrometry were not subjected to further analysis if they were either absent in less than 80% of the samples, or they were present in concentrations that were too low or exhibited poor spectral quality, making it challenging to identify them as metabolites. From the comprehensive analysis, a consistent and confident detection was achieved for a total of 237 endogenous metabolites. To validate the significance of the identified metabolites in distinguishing different classes, those with a VIP score exceeding 1.5 were subjected to confirmation using independent analytical standards (corresponding to MSI level 1).

### 2.4. Statistical Analysis

#### 2.4.1. Animals’ Characteristics

The statistical analysis of the characteristics of the subjects included in the study was conducted utilising R, a programming language and environment dedicated to statistical computing and data visualization [37]. To assess the normal distribution of the data, the Shapiro–Wilks test was employed. Given that the data exhibited a normal distribution, the one-way analysis of variance (ANOVA) with Tukey’s post hoc test was applied for making inter-group comparisons. The significance level (alpha) was set at 0.05 to determine statistical significance.

#### 2.4.2. Metabolomic Data Analysis

The GC–MS metabolomics results were compiled into a matrix file with comma-separated values, which was then loaded into specialized software (MetaboPredict^®^, Theoreo srl, Montecorvino Pugliano, Italy) for statistical analysis. The results were compiled into a comma-separated matrix file and imported into the appropriate software for subsequent statistical analysis. Prior to analysis, the chromatographic data underwent several preprocessing steps. Data alignment was achieved using the Parametric Time Wrapping algorithm [38] followed by peak picking, integration, and deconvolution. The resulting chromatographic data were organized in a tabular format, with each sample represented by a row, and each metabolite represented by a column. Normalization procedures were applied to the data, involving both data transformation and scaling. The data transformation employed a generalized logarithmic transformation, while the scaling was performed using an auto-scaling approach, which involved mean-centring and dividing each variable (metabolite) by its standard deviation. Additionally, normalization was performed using the chromatographic peak area of the internal standard and the exact sample weight [39].

Analysis was conducted categorizing the samples based on the treatment, resulting in five classes: Cadmium, Copper, Nickel, Mix, and CTRL. In order to improve the reliability of the data analysis, we employed the SMOTE [40] algorithm to generate five synthetic samples, one for each class.

Class separations were further explored using the Partial Least Square Discriminant Analysis (PLS-DA), a supervised method that utilises multivariate regression techniques to identify linear combinations of the original variables (X) that are predictive of class membership (Y). A permutation test was performed to determine the significance of class discrimination. In each permutation, a PLS-DA model was built between the data (X) and permuted class labels (Y), using the optimal number of components determined by cross-validation based on the original class assignment. Two types of test statistics were employed to assess class discrimination. The first was based on prediction accuracy during training, while the second utilised the separation distance calculated as the ratio between the sum of squares “Between” groups and the sum of squares “Within” groups (B/W-ratio). If the observed test statistic fell within the distribution derived from permuted class assignments, the class discrimination was considered statistically insignificant [41]. Variable Importance in Projection (VIP) scores were computed for each component in the PLS-DA analysis. The VIP represents a weighted sum of squares of the PLS loadings, considering the amount of explained Y-variation in each dimension.

## 3. Results

The results were obtained by analysing sperm pellets from 10 mussel samples. These samples included 2 treated with cadmium, 2 with copper, 2 with nickel, 2 treated with a mixture of these metals, and 2 untreated controls (CTRL). In order to improve the reliability of the data analysis, we employed the SMOTE algorithm to generate five synthetic samples, one for each class.

**Figure 1:**
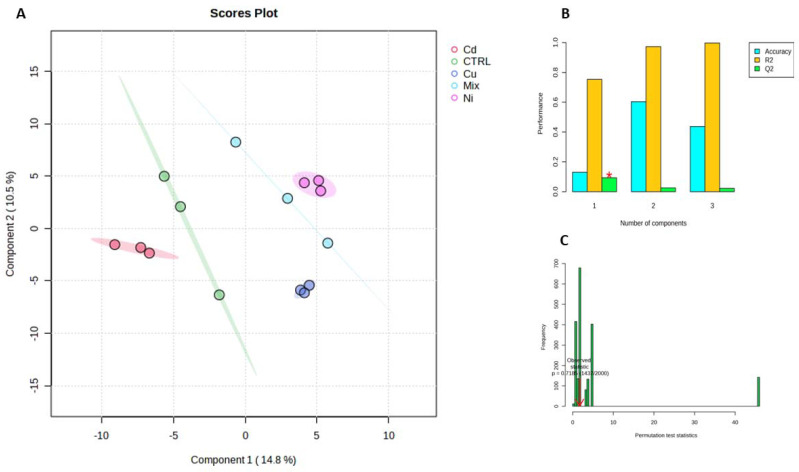
Partial least square discriminant analysis (PLS-DA) used to discriminate the gamete samples of mussels treated with cadmium (Red), copper (Blue), mix (Cyan), nickel (Violet) and CTRL (Green). Each axis is accompanied by the percentage of variance explained, indicated within parentheses (**A**). Panel (**B**) presents the classification performance of the PLS-DA model, with an escalating number of latent variables. The superior classifier is denoted by a red star. In (**C**), the permutation test results are displayed, where models were constructed by randomly assigning class labels. The performance of these models was then compared to that of the original model, built with the correct class assignments. The red arrow indicates the performance of the model built with the non-permutated classes.

**Figure 2:**
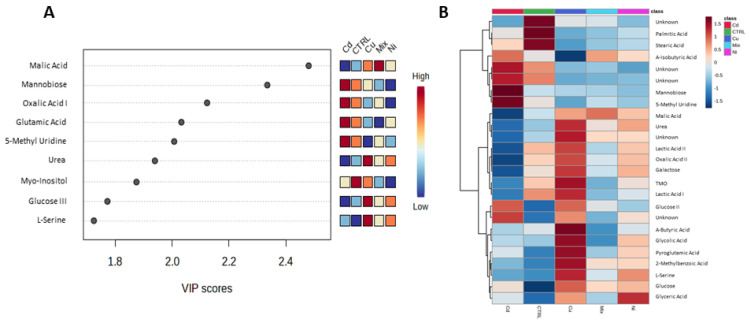
(**A**) Metabolites relevant to class separation (VIP score > 1.5). (**B**) Heatmap displaying the metabolites’ selected by ANOVA concentrations. The utilisation of cluster analysis facilitated the identification of four distinct clusters of metabolites, determined by their average concentration levels within the five classes.

A PLS-DA algorithm was applied on the gamete dataset, resulting in a non-overfitted and statistically significant model with a good class separation (R^2^ = 0.773, Q^2^ = 0.119, cross-validation accuracy = 0.153, and 1 latent variable, *p* = 0.719), as shown in Figure 1 (Panels A, B, and C). Again, metabolites with a VIP score > 1.5 were identified (Figure 2A), and a heatmap was used to collectively represent the top 25 metabolites that emerged from the ANOVA (Figure 2B).

**Figure 3:**
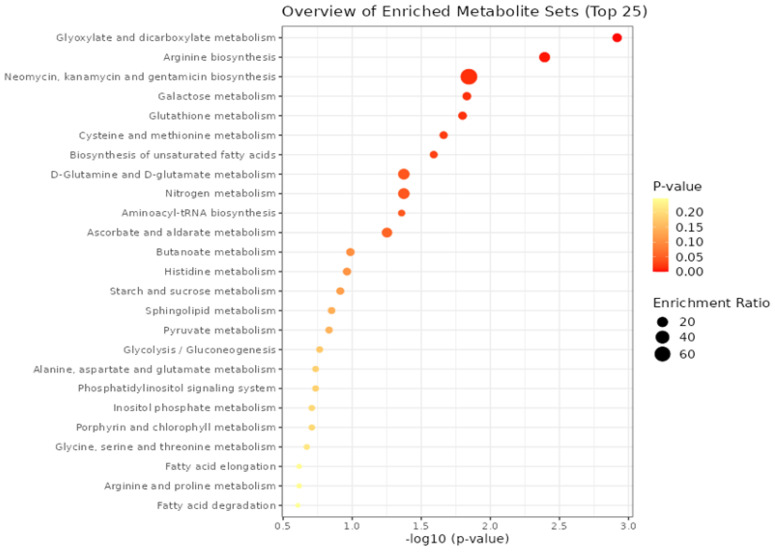
Enrichment analysis from spermatozoa datasets.

In addition, the detected metabolites were employed in a metabolite-set enrichment analysis, as depicted in Figure 3. This analysis uncovered intricate interactions within various metabolic pathways and metabolites.

## 4. Discussion

The immoderate utilisation of heavy metals has not only resulted in a massive increase in these substances in terrestrial but also in aquatic environments [42]. Metabolomics represents an advanced approach to the assessment of the health status of organisms through the identification of low-molecular-weight metabolites, the production and levels of which change in response to the physiological, developmental, or pathological state of cells, tissues, organs, or entire organisms [12]. Environmental metabolomics may be utilised as a real-time means of monitoring, which can also establish the mechanisms of change or identification of the specific stressor [16]. In an effort to measure the responses of the Mediterranean *mussels Mytilus galloprovincialis* to environmental changes, in recent decades, metabolomics has proved to be a valuable and high-performance approach. In the present study we identified 237 metabolites in the spermatozoa of *M. galloprovincialis* and quantified them after exposure of this organism to different metals and to their mixture. In particular, the most abundant metabolites found in spermatozoa were eight (malic acid, mannobiose, oxalic acid I, glutamic acid, 5-methyl uridine, urea, myo-inositol, glucose III, and L-serine). The level of these metabolites varied, following exposure to the different metals tested in this study. The malic acid (MA) amount increased after exposure of mussels to copper and nickel, but particularly with the exposure to the mixture of the metals tested in this study compared to the control condition, while it decreased with respect to the control condition after exposure to cadmium. MA is an excellent factor for chelating toxic elements [43]. MA has been shown to be able to increase the expression of genes involved in antioxidant enzymes. An example case involves *M. sacchariflorus*, when exposed to stress induced by cadmium at a concentration of 100 µM CdCl_2_, exhibited an elevated expression of key antioxidant enzymes, namely Cu/Zn-SOD, POD1, glutathione peroxidase (GPX1), glutathione S-transferase (GST1), monodehydroascorbate reductase (MDHAR), and dehydroascorbate reductase (DHAR) when treated with either Cd alone or MA alone (at 100 µM each). Moreover, the simultaneous application of MA and Cd further stimulated the expression of Cu/Zn-SOD, POD1, GR1, GPX1, and GST1, all of which play a crucial role in protecting against oxidative stress. Remarkably, the combined treatment led to a substantial up to 4.7-fold increase compared to Cd-control plants [44].

Similarly, in the context of rice Os-Nramp1, OsIRT1 (iron-regulated transporter), OsHMA3, and OsNAS1 (nicotianamine synthase), exposure to Cd stress at a concentration of 25 µM CdCl_2_ resulted in reduced expression of these genes [45].

Mannobiose, oxalic acid, glutamic acid, and 5-methyl-uridine, on the other hand, were found to be present in greater quantities after exposure to cadmium than in the control, where they were present at average values, whereas under all other exposure conditions, there was a decrease in the presence of these metabolites. The increase in oxalic acid following exposure to cadmium is in line with a study conducted on the fungus *Phanerochaete chrysosporium* in which the role of oxalic acid in Cd uptake and participation in the detoxification process was examined. The formation of a metal–oxalate complex may thus provide a detoxification mechanism through its effect on metal bioavailability, whereby many fungi can survive and grow in environments containing high concentrations of toxic metals [46]. In addition, oxalic acid correlated significantly with the number of sperm head anomalies and the pH value [47]. Glutamate also alleviates cadmium toxicity, as reported in rice via suppressing cadmium uptake and translocation [48]. Glutamate has a significant role in metabolism, that is to produce ATP as an energy source for spermatozoa motility [41]. Glutamic acid is necessary for spermatozoa metabolism; the decrease in glutamic acid level results in a reduction in spermatozoa motility [49]. Glutamic acid plays a pivotal role in maintaining the quality of spermatozoa, particularly by safeguarding their plasma membrane against damage caused by lipid peroxides. This mechanism acts as an antioxidant defense to protect cells from the harmful effects of free radicals. Moreover, nitric oxide plays a significant role in neutralizing the superoxide generated during spermatozoa’s oxygen consumption process. Excessive superoxide levels can impair the phospholipid membrane of spermatozoa, leading to functional deficits.

However, glutamic acid counteracts this issue by promoting the production of nitric oxide, which in turn prevents the accumulation of lipid peroxides in the spermatozoa’s membrane. By doing so, glutamic acid acts as a protective agent, ensuring the integrity of the spermatozoa’s essential structures.

Furthermore, the glutamic acid acts as a facilitator in the process of sugar fractionation within spermatozoa, which positively impacts their metabolism and enhances energy availability. This, in turn, regulates the spermatogenesis process within the testes, resulting in the production of higher-quality spermatozoa, particularly in terms of morphology [50].

With regard to urea, it was more present than the control after exposure to nickel, copper, and the mixture, with a maximum occurring after exposure to copper. With regard to cadmium exposure, urea was present in lower amounts than the control. The osmolality and pH of the media remained unaffected by the presence of urea. However, elevated concentrations of urea beyond normal physiological levels had detrimental effects on critical aspects of sperm functionality, such as sperm membrane integrity, mitochondrial membrane potential, and acrosome integrity, all of which play crucial roles in the successful process of fertilization. These findings were documented in a study cited as [51]. Furthermore, there was a significant and negative correlation between the concentration of ammonia in the seminal fluid and sperm motility (*p* < 0.05). Urea and creatinine levels in the seminal fluid also displayed a negative correlation concerning sperm count (*p* < 0.05). Additionally, an inverse correlation was observed between urea and uric acid levels in the seminal plasma and sperm morphology (*p* < 0.05). Moreover, a significant negative correlation emerged between seminal uric acid and urea concentrations and the percentage of fertilization rate (*p* < 0.05).

In conclusion, as reported by Allahkarami et al. [52] elevated levels of urea and uric acid in seminal plasma exert an adverse influence on the fertilization rate, indicating their potential role as determinants of impaired fertility.

In contrast, myo-inositol was found to be the most abundant in the control condition compared to all other exposure conditions, particularly after nickel exposure. Among all, myo-inositol (myo-ins) represents a safe compound that proved useful in issues related to fertility and pregnancy. In fact, myo-ins participates in several signalling processes, including the pathways of insulin and gonadotropins, and, therefore, it is likely to positively affect fertility. Recent investigations have highlighted the significant involvement of myo-Inositol in the maturation of male gametes and their migration from the epididymis [53]. Notably, to combat the detrimental effects of reactive oxygen species (ROS), emerging therapeutic approaches have explored the potential of myo-Inositol. Noteworthy data from recent studies have demonstrated that myo-Inositol enhances spermatozoa motility. Specifically, a meticulous ultrastructural analysis, utilising both scanning and transmission electron microscopy, revealed a reduction in amorphous fibrous material surrounding spermatozoa, along with improved mitochondrial cristae morphology [54]. Beyond this remarkable investigation, substantial evidence further substantiates the role of myo-Inositol in modulating the molecular and cellular pathways that govern the physiological regulation of sperm mitochondrial functions and DNA integrity [55].

Finally, glucose and L-serine, were found to be more present after exposure to copper, nickel, and the mixture, compared to the control condition. This could be explained, considering that serine is considered a non-essential amino acid that provides critical biological activities, which range from protein synthesis to cell signalling, the latter principally via post-translational modification by phosphorylation [56,57]. Serine is a precursor of glycine and cysteine and can be utilised to synthesise glutathione. Synthesising glutathione, in order to decrease oxidative stress and to enhance activity, increases the activity of antioxidant enzymes [58,59]. On the other hand, the exposure to these metals and to the mixture also caused an increase in PARP expression (manuscript under review into Chemico–Biological Interactions). In conclusion, the metabolomics, the youngest omics technology, represent a promising new ‘omics’ approach for profiles and identify biomarkers that are indicative of the physiological responses of organism (e.g., whole organisms, tissue, or cells) both under natural and controlled conditions. This technology can qualitatively and quantitatively reflect the impact of internal and external factors on the metabolism of organisms, and thus offers a snapshot of used biochemical procedures as an effect of the environment. Gaining increasing application in aquaculture, metabolomics opens the possibility to evaluate several important factors or key issues across the aquaculture value chain. In particular, our results can be useful in some of these applications such as ecotoxicology, health and disease, and environmental monitoring. Furthermore, these results from metabolomics could make contributions in both nutrition and ecotoxicology studies to track the freshness of mussels and investigate the influence that environmental changes may have. After all, the Mediterranean mussel *M. galloprovincialis* (Lamarck 1819) is a widespread mussel usually present in the human diet and is commonly employed in environmental monitoring programmes around the world as a bio-indicator. Furthermore, in recent years, metabolomics is emerging as a very useful technology in both nutrition and ecotoxicology studies, and can identify often unexpected problem or risk areas through a broad analysis of biological conditions and the possible areas of risk [60].

## 5. Conclusions

The findings of this study shed light on the impact of heavy metal exposure on the metabolic profile of *Mytilus galloprovincialis* spermatozoa. The investigation revealed alterations in the levels of the eight most abundant metabolites, which play crucial roles in various pathways essential for sperm function. These preliminary results already raise concerns about the potential negative effects of escalating heavy metal pollution in our oceans. The metabolic changes in spermatozoa were analysed using a metabolomics approach based on GC–MS analysis. Statistical methods, including PLS-DA, were employed for data analysis, effectively separating classes and identifying key metabolites through VIP scores. Heatmaps and cluster analysis further evaluated the metabolites, while metabolite-set enrichment analysis revealed complex interactions within metabolic pathways.

This pioneering research represents the first exploration into the metabolic changes occurring in *Mytilus galloprovincialis* spermatozoa when exposed to heavy metals. It serves as a significant steppingstone, emphasizing the need for further investigations with greater molecular resolution to fully comprehend the implications of these initial alterations.

## Data Availability

The data presented in this study are available upon request from the corresponding authors. The data are not publicly available because the corresponding author can maintain and verify the integrity and quality of the data requested with each request, keep track of all possible accesses, and have direct contact with those requesting the data to resolve any doubts or concerns.
